# A Study on the Effects of Lateral-Wedge Insoles on Plantar-Pressure Pattern for Medial Knee Osteoarthritis Using the Wearable Sensing Insole

**DOI:** 10.3390/s23010084

**Published:** 2022-12-22

**Authors:** Wei-Ching Hsu, Li-Wei Chou, Hsiao-Yen Chiu, Chang-Wei Hsieh, Wen-Pin Hu

**Affiliations:** 1 Department of Bioinformatics and Medical Engineering, Asia University, Taichung City 41354, Taiwan; 2 Department of Physical Medicine and Rehabilitation, Asia University Hospital, Taichung City 41354, Taiwan; 3 Department of Physical Medicine and Rehabilitation, China Medical University Hospital, Taichung City 40402, Taiwan; 4 Department of Physical Therapy, Graduate Institute of Rehabilitation Science, China Medical University, Taichung City 40402, Taiwan; 5 Department of Computer Science & Information Engineering, Asia University, Taichung City 41354, Taiwan

**Keywords:** disease, knee osteoarthritis, lateral wedge insole, plantar pressure, pressure-sensing insole

## Abstract

Patients with knee osteoarthritis have a unique plantar-pressure pattern during walking, and lateral-wedge insoles are one of the treatment options. Participants were randomly assigned to either the lateral-wedge insole group or the ordinary insole group. The Visual Analog Scale (VAS), Western Ontario and McMaster Universities Osteoarthritis Index (WOMAC), and plantar-pressure test scores were evaluated at the baseline and at 20 weeks. Plantar pressure data were collected using a pressure insole with 89 sensing locations. In the ordinary insole group, the function and total WOMAC scores decreased significantly (function score, 24.8 (baseline) to 16.5 (week 20); total score, 34.9 (baseline) to 24.6 (week 20)). During walking, the transverse width of the center of pressure as a percentage of foot width (%Trans) significantly increased in the ordinary insole group (baseline, 6.3%; week 20, 14.8%). In addition, the values of partial foot pressure as a percentage of body weight (%PFP) on the forefoot (baseline, 30.3%; week 20, 39.2%) and heel (baseline, 28.1%; week 20, 16.9%) also increased significantly in the ordinary insole group. Significant group-by-time interaction effects were observed for partial foot pressure per body weight in the forefoot (*p* = 0.031) and heel (*p* = 0.024). In the ordinary insole group, the plantar pressure on the heel significantly decreased (*p* = 0.011) and that on the forefoot significantly increased (*p* = 0.023). In contrast, plantar pressure remained stable in all regions in the lateral-wedge insole group. Thus, lateral-wedge insoles may protect against plantar pressure deterioration in patients with knee osteoarthritis.

## 1. Introduction

Knee osteoarthritis (OA) often causes knee pain and stiffness in the knee joints [1]. In addition, some studies [2,3] have reported that patients with knee OA demonstrate a unique foot pressure pattern while walking. For example, partial foot pressure as the percentage of body weight (%PFP) in the hallux and heel regions during walking were significantly lower in patients with OA than in the healthy population [3]. In contrast, the %PFP in the central region was higher in such patients [3]. These consequences cause forward displacement of the center of pressure (COP) at the initiation of walking, and backward displacement of the COP at the end of walking. Moreover, they decrease the anteroposterior (AP) length of the COP path measured and is expressed as a percentage of the foot length (%Long), as well as the transverse width of the COP path measured from the most medial to the most lateral point of the COP path and is expressed as a percentage of the maximum foot width (%Trans).

Treatment with lateral-wedge insoles is a nonsurgical method for knee OA [4]. To the best of our knowledge, Sasaki and Yasuda [5] were the first to use lateral-wedge insoles to improve pain severity and function in patients with knee OA. Their central hypothesis was based on decreasing loading in the medial tibiofemoral compartment. However, although subsequent studies proved that lateral-wedge insoles can decrease knee adduction moment [6], there is no definite conclusion regarding the results of pain and functional improvement [7,8,9,10].

Foot pressure measurement is widely used to assess lower limb disease or exercise training. These measurements involve two main methods: a pressure plate and a pressure-sensing insole. Lidtke et al. [11] used a pressure plate to measure foot pressure characteristics in patients with knee OA. They found that the COP deviated to the medial side of the foot in such patients compared with those in the control group, indicating that the foot pressure distribution in patients with knee OA during walking differed from that observed in healthy individuals. The F-Scan pressure sensor system, introduced in 1998, is currently commonly used in studies [12]. Saito et al. [3] used the F-Scan II system to conduct a series of studies on knee OA. Another commonly used sensor insole is the Pedar-X insole system introduced in 2009. Leitch et al. [13] used this system to evaluate the effects of lateral-wedge insoles on foot pressure in patients with knee OA. They observed increased lateral heel pressure and anterolateral displacement of the COP in both patients and healthy individuals.

Zhang et al. [14] measured plantar pressure during walking in 23 women with knee OA and 23 women without knee OA without investigating the effect of lateral-wedge insoles. They characterized plantar loading of participants with and without knee OA. Compared with women without knee OA, women with knee OA showed more extensive plantar loading at the midfoot, first metatarsophalangeal joint, and second metatarsophalangeal joint. Increased plantar loading may cause foot pronation and change their gait during walking. Tse et al. [15] measured the in-shoe plantar pressure during walking with lateral-wedge insoles in a sample of healthy individuals. They reported that appropriate lateral-wedge insole variation might prevent the exacerbation of concomitant foot symptoms. Other related studies recruited participants to study the effects of lateral-wedge insoles using gait analysis [16,17]. In these studies, participants with a biomechanical response to wedges could be recognized based on gait analysis. However, evaluations in gait laboratories are expensive and not widely available.

Long-term follow-up studies of the effects of lateral-wedge insoles on foot pressure in patients with knee OA are limited. Therefore, this randomized double-blind study with a 20-week follow-up was conducted. Although several studies have evaluated the effects of lateral-wedge insoles on pain, function, or biomechanical parameters, no clinical trial has demonstrated the long-term influence of lateral-wedge insoles on plantar pressure in patients with knee OA. Therefore, this study aimed to determine the clinical effects of 20 weeks of insole dressing in patients with knee OA and hypothesized that lateral-wedge insoles prevent deterioration of plantar-pressure patterns. To the best of our knowledge, this is the first study to evaluate the long-term (20 weeks) effect of lateral-wedge insoles on patients with knee OA using a pressure-sensing insole.

## 2. Materials and Methods

### 2.1. Recruitment and Eligibility

This study was conducted from August 1, 2020, to December 31, 2021. All participants were recruited from the outpatient clinic of Asia University Hospital, Taichung, Taiwan. The inclusion criteria were as follows: (1) age ≥ 55 years; (2) pain in the medial knee; and (3) Kellgren–Lawrence grades 2–4 for knee OA on the affected side [18]. The exclusion criteria were as follows: (1) inability to walk independently and requiring a walking aid; (2) history of central nervous system disease such as stroke, Parkinson’s disease, Alzheimer’s disease, and multiple sclerosis; (3) rheumatoid arthritis or related lower-extremity inflammatory joint diseases such as gout; (4) history of severe trauma or fracture in the lower extremities, or surgery on the lower extremities, such as joint replacement (hip, knee, and ankle), amputation, or ligament repair; and (5) suspected local or systemic infection. This study was approved by the Research Ethics Committee of the China Medical University and Hospital, Taichung, Taiwan (protocol code: CMUH109-REC1-219). All participants provided written informed consent. All collected data were anonymized before analysis to ensure confidentiality.

### 2.2. Study Design and Intervention

**Figure 1:**
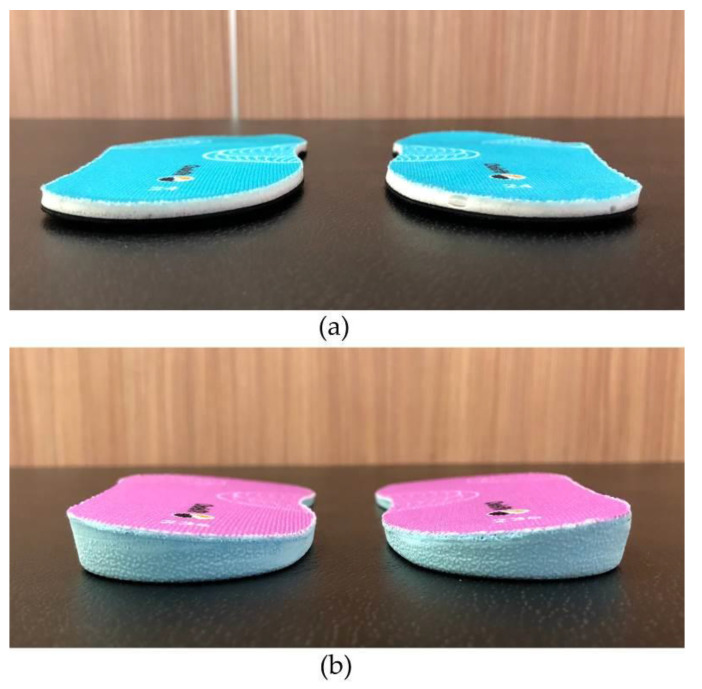
Two types of functional insoles. (**a**) Ordinary insoles with arch support (**b**) Lateral-wedge insoles with arch support.

Participants meeting the inclusion criteria were randomly assigned to the control or experimental groups. The participants in the experimental group received a pair of lateral-wedge insoles with arch support. A pair of ordinary insoles with arch support was allocated to each participant in the control group. Figure 1 shows the ordinary and lateral-wedge insoles. Both types of insoles were manufactured with non-woven thermoplastic material (thermoplastic polyurethane) and polyurethane, and modified to fit the foot arch using the foot assessment system developed by Choisfit (Taipei City, Taiwan) [19]. An occupational therapist at Asia University Hospital helped create the insoles for the two groups, but did not participate in the experiments and follow-up activities. The ordinary insoles could provide arch support, while the lateral-wedge insoles provided arch support and had a 6° lateral inclination. Both types of insoles were placed in shoes owned by the participant and worn daily.

All participants underwent tests at week 0 for baseline measurements before dressing insoles. After testing at week 0, participants were asked to use the insoles for at least 4 h per day. During the experimental period, drug injection into the knee joint or surrounding soft tissue and interventional treatment, such as extracorporeal shock waves or nerve blocks, were not allowed. Oral non-steroidal anti-inflammatory drugs could be used to relieve pain, if necessary. The participants underwent tests again at week 20.

### 2.3. Evaluations and Measurement

The participants underwent tests at weeks 0 and 20. The testing items included the Visual Analog Scale (VAS), Western Ontario and McMaster Universities Osteoarthritis Index (WOMAC), and plantar pressure measurements.

VAS is widely used clinically to evaluate musculoskeletal pain in patients [20]. It uses a 10-cm straight line with the intensity of pain oriented from the left (no pain) to the right (worst pain). Participants were asked to mark the level with a vertical line, and the length from the left end to the vertical line was measured to express the pain level of each participant.

WOMAC was proposed in 1981 [21] as a standardized questionnaire to assess the condition of people with OA of the knee and hip. This questionnaire is divided into three parts: pain, stiffness, and physical difficulty in joint function to evaluate the structure and function of the hip and knee joints. Each item ranges from 0 (no pain, stiffness, or difficulty) to 4 (severe pain, stiffness, or difficulty). The pain, stiffness, and joint function sections have 5, 2, and 17 items, respectively. Therefore, the maximum score for the questionnaire is 96. The score obtained for each section can be partially compared or converted into a percentage of the total score for comparison. Several studies have shown that WOMAC has objective reliability, validity, and sensitivity in the assessment of knee OA [22,23,24]. Moreover, it is a widely employed self-assessment tool for patients with degenerative arthritis.

**Figure 2:**
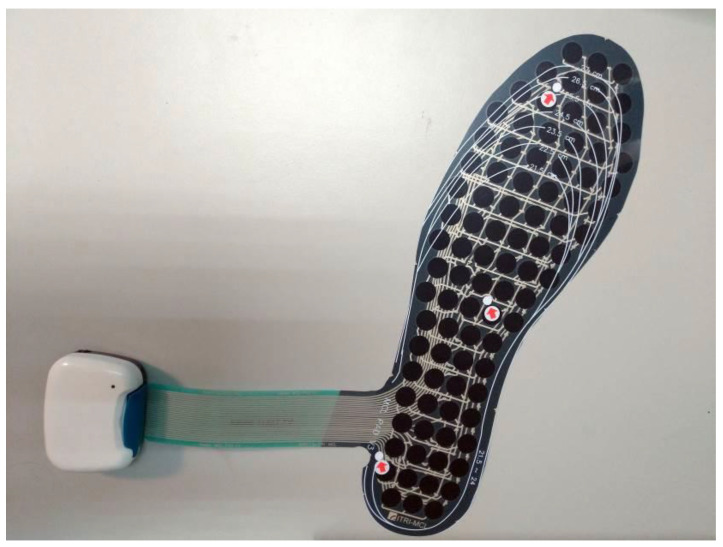
The pressure-sensing insole contains 89 sensing spots.

**Figure 3:**
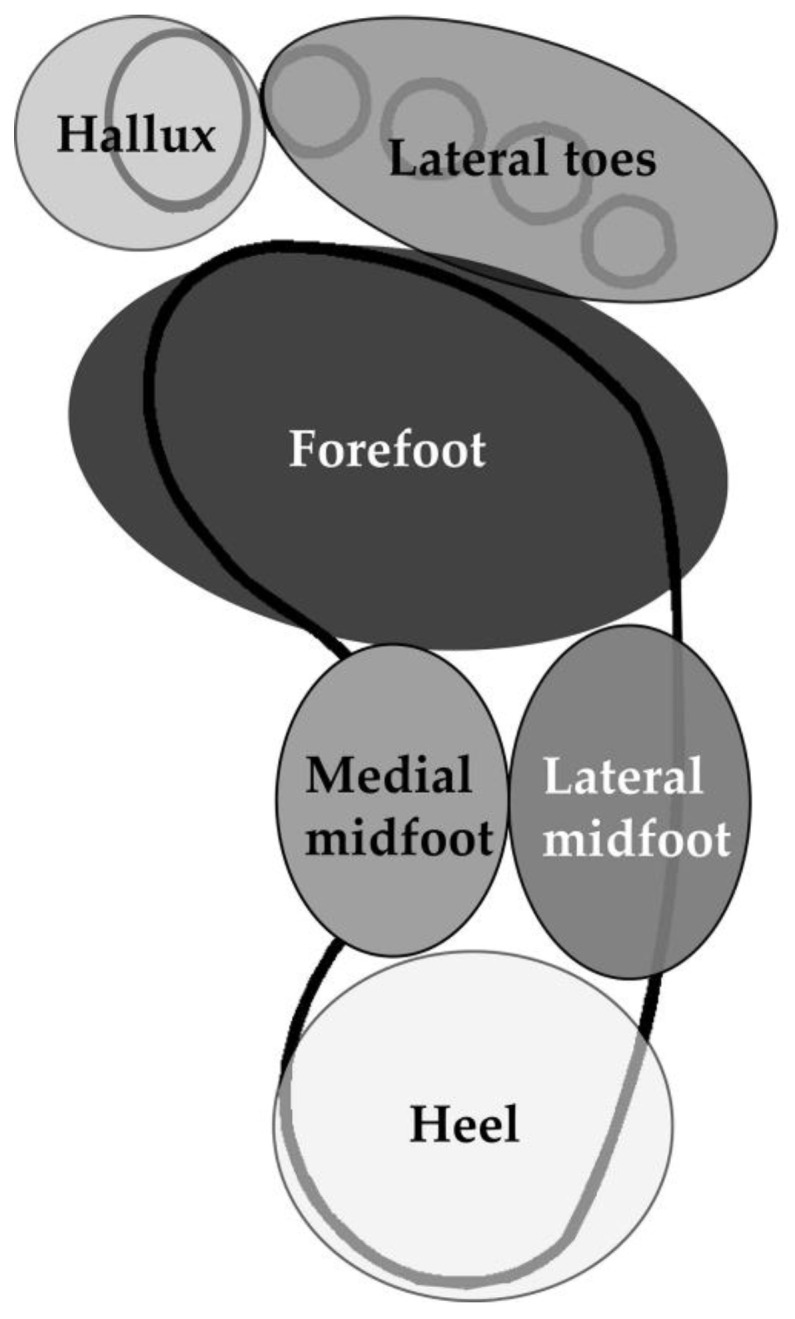
A scheme of the areas for calculating the percentages of pressure distribution in the stance phase of gait for the affected side of osteoarthritis.

All participants underwent plantar pressure measurement at weeks 0 and 20. During the test, the participants were asked to wear shoes prepared by the researchers, and were allowed to practice for 5 min before performing the specified activities. Each participant had to perform two activities: (1) quiet standing with a natural hip-width stance, feet parallel, and eyes open for 30 s; (2) walking 10 m back and forth on flat, unobstructed ground for a total length of 20 m. This study used a pressure-sensing insole fabricated by the Industrial Technology Research Institute (ITRI, Hsinchu County, Taiwan) [25]. The specifications of the pressure-sensing insole are listed in Table S1 (Supplementary Material). Figure 2 shows the entire device, which contains 89 sensing spots and a white box with a wireless transmission unit and a battery inside. The sampling rate of this device was 30 Hz, the diameter of each spot was 10 mm, and the data were transmitted to a laptop via Bluetooth. Each sensing spot had an excellent linear trend of the output signal and pressure in the range 0–40 psi, and the pressure was digitized as a value between 0 and 255. The characteristics of the sensing spots were identical. This insole was placed into the shoe above the original insole of the shoe, and the bottom of the insole was fixed using double-sided tape to avoid slippage between the insoles. Data were analyzed using MATLAB programs (MathWorks, Inc., Natick, MA, USA) written by our group. The COP displacements in the mediolateral and AP directions were analyzed based on data recorded during quiet standing. In addition, %Long and %Trans were analyzed using walking data. Further, the percentage pressure distribution in the hallux, lateral toes, forefoot, medial midfoot, lateral midfoot, and heel for the affected side were calculated during the stance phase of gait. The COP displacement, percentage of the COP path, and percentage of partial foot pressure were calculated using the equations shown in the Supplementary Materials (Equations (S1)–(S7)). Figure 3 shows the defined areas for calculating the percentage pressure distribution during walking.

### 2.4. Data Analysis

The chi-square test was used to analyze sex. The Shapiro–Wilk test was used to determine whether continuous variables were normally distributed. The t-test was used to compare the univariate analysis of continuous variables, including age, body weight, and body mass index. Repeated measures analysis of variance (RMANOVA) was used to analyze the group-by-time interaction effect for VAS, WOMAC, and plantar pressures. The within-participant factor was the time of the test (0 and 20 weeks). The between-participants factor was the difference in the intervention (lateral-wedge insole and ordinary insole). RMANOVA was also employed to compare continuous variables within the same group at different time points with the Bonferroni correction. RMANOVA was suitable for tracking and comparing participants’ progress at the beginning and at other time points. MedCalc 20.015 (MedCalc Software, Ostend, Belgium) was used for statistical analyses. Statistical significance was defined as *p* < 0.05.

## 3. Results

### 3.1. Participant Characteristics

**Figure 4:**
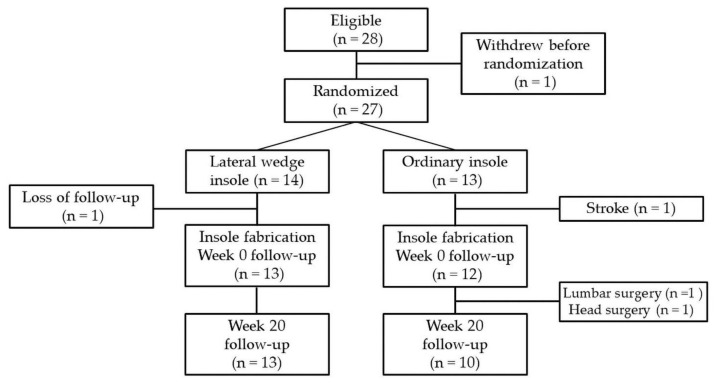
Study flow diagram for the lateral-wedge insole group and ordinary insole group.

Overall, 28 patients were included in this study (Figure 4). Of these, one patient withdrew before randomization, and the remaining 27 patients were randomized into the lateral-wedge (experimental group, n = 14) and ordinary (control group, n = 13) insole groups. Of the 14 patients in the lateral-wedge insole group, one was lost to follow-up before the baseline measurements, and 13 completed the evaluations at 0 and 20 weeks. Of the 13 patients in the ordinary insole group, one dropped out due to stroke before the baseline evaluation, and two participants dropped out due to lumbar surgery and head surgery before the week 20 evaluation; the remaining ten patients completed both evaluations. There were no significant differences in age, sex, weight, and body mass index between the two groups (Table 1).

### 3.2. Outcome Measurements

In this section, the statistical results of RMANOVA for the VAS (Table 2), WOMAC (Table 3), and plantar pressure (Table 4, Table 5 and Table 6) are presented. Significant group-by-time interaction effects were observed for the WOMAC function scores (*p* = 0.031) (Table 3) and the partial foot pressure per body weight for the forefoot (*p* = 0.031) and heel (*p* = 0.024) (Table 6).

Regarding the VAS scores, Table 2 shows no significant group-by-time effect. Participants in both groups completed the WOMAC questionnaire at weeks 0 and 20, and the data were used for statistical analysis, which was performed to evaluate the pain, stiffness, function, and total scores. Table 3 shows the average data and the results obtained using RMANOVA. Regarding the WOMAC function scores, a significant decrease (*p* = 0.035; for the superscript letter ‘a’) was identified in the ordinary insole group at week 20 compared with the baseline, whereas no significant change was observed in the lateral-wedge insole group. The results revealed an improvement in the WOMAC function scores in the ordinary insole group, but not in the lateral-wedge insole group. Regarding the WOMAC total scores, a significant decrease (*p* = 0.030; for the superscript letter ‘b’) was observed in the ordinary insole group, but no significant group-by-time effect was observed.

During quiet standing, the AP displacement of the COP (Table 4) in the ordinary insole group significantly increased at week 20 compared to the baseline (*p* = 0.008; for the superscript letter ‘a’). There was no significant difference in AP displacement between the two time points in the lateral-wedge insole group. In addition, Table 5 shows that %Trans during walking significantly increased in the ordinary insole group at week 20 (*p* = 0.007; for the superscript letter ‘a’), but not in the lateral-wedge insole group. Additionally, no significant group-by-time interaction effects were observed.

The percentage of partial foot pressure distribution in the hallux, lateral toes, forefoot, medial midfoot, lateral midfoot, and heel during walking was compared between the baseline and week 20 (Table 6). In the ordinary insole group, the partial foot pressure distribution showed a significant increase (*p* = 0.023; for the superscript letter ‘a’) on the forefoot and a significant decrease (*p* = 0.011; for the superscript letter ‘b’) on the heel at week 20 compared with that at the baseline. In the lateral wedge insole group, no significant change in the partial foot pressure distribution at week 20 was observed compared with that at the baseline. Because no significant changes were observed in the partial foot pressure distribution on the forefoot and heel, we suggest that the lateral-wedge insoles could help slow the deterioration of the joint function in patients with OA.

## 4. Discussion

This study aimed to compare the effects of lateral-wedge and ordinary insole treatment on knee OA. The results revealed significant group-by-time effects on WOMAC function, and partial foot pressure per body weight on the forefoot and heel. Although, the ordinary insole group showed a significant decrease in WOMAC total scores; both the AP displacement of the COP during quiet standing and %Trans increased, no group-by-time interaction effects were observed for these parameters.

Parkes et al. [9] reported a meta-analysis of 12 clinical trials. Their results indicated that the lateral-wedge insole could improve pain severity in the knee OA group compared with the control group (placebo treatment, including using a neutral or flat insole shoe). However, when the control group was reclassified according to treatment, there was no significant difference in pain alleviation between the lateral-wedge and neutral insoles. In addition, Zhang et al. (2018) [10] published a meta-analysis of randomized controlled trials that included ten clinical studies; no intervention was conducted in the control group in two studies, and neutral insoles were used in the control group in eight studies. The results showed that pain and functional improvement in the lateral-wedge insole group were not better than those in the control group. Hunt et al. [26] conducted a study involving patients with medial knee OA and pronated feet. Participants who used lateral-wedge insoles with custom foot arch support had significantly improved knee and foot pain and function based on WOMAC scores. However, participants who used lateral-wedge insoles without custom foot arch support did not improve effectively. According to these studies, lateral-wedge insoles had no superior effect on subjective parameters compared with other types of insoles, and the arch support design might improve symptoms in patients with knee OA. Our study showed similar results for the VAS and WOMAC scores. The ordinary insole group showed significantly decreased WOMAC function and total scores, while the lateral-wedge insole group showed no significant change in VAS or WOMAC scores [26]. However, a 3D motion-capture system and force platforms were adopted to investigate walking kinematics and kinetic data from patients with OA [27]. In previous studies, participants were tested randomly using lateral-wedge insoles with six different angulations (0, 2, 4, 6, 8, and 10°). The findings demonstrated that lateral-wedge insoles > 2° could significantly reduce the load on the medial side of the knee, which might delay OA progression or even improve the clinical condition. Our findings also revealed that lateral-wedge insoles with arch support and a 6° lateral inclination could prevent the deterioration of plantar pressure patterns.

Patients with knee OA have a typical plantar-pressure pattern during walking, and the deterioration can be recovered with invasive treatments. Saito et al. [2] evaluated changes in patients with knee OA before and after total knee arthroplasty and classified them into the no foot pain, resolved foot pain, and remaining foot pain groups. They found that %PFPs in the medial heel, lateral heel, hallux, and lateral toe regions increased significantly in the no foot pain and resolved foot pain groups. Moreover, %Long and %Trans improved significantly after surgery. In the remaining foot pain group, only %PFP in the medial heel region and %Long improved after surgery, and there were no significant changes in the other items.

In our study, the ordinary insole group had a plantar-pressure pattern typical for knee OA during 20-weeks of follow-up. Plantar-pressure measurements revealed a decrease in %PFP on the heel and an increase in %PFP on the forefoot in the ordinary insole group. While plantar pressure shifting to the central foot areas was observed in the ordinary insole group, the lateral-wedge insole group showed no significant change in plantar pressure. Our results also revealed increases in %Long and %Trans for both groups, but only the %Trans for the ordinary insole group was significantly different. Additionally, the group-by-time interaction effect was not significant for %Long and %Trans. However, the relatively small number of cases in our study might have limited the ability to detect this trend. In brief, our results suggest that lateral-wedge insoles do not have the same effect as invasive procedures but might prevent disease progression in patients with knee OA.

Based on a meta-analysis of 27 studies, Shaw et al. [6] indicated that although the lateral-wedge insole could decrease the knee adduction torque, it might increase the ankle eversion angle. If arch support is increased, it would reduce the knee adduction torque, but it could decrease the effects of the insole on the ankle. In addition, several recent studies [28,29] have reported that gait biomechanics, the ankle and foot, and subjective symptoms must be evaluated when designing insoles for patients with knee OA. This can ensure that appropriate insoles are prescribed, and better outcomes are achieved if early intervention is performed.

This study had some limitations. First, the study design only considered the diagnosis of knee OA during enrollment and did not evaluate the ankle and foot symptoms. According to recent studies, ankle and foot symptoms are important factors that affect the overall lower limb biomechanics. Therefore, future studies should record ankle and foot conditions to better analyze the treatment effects. Second, the type of footwear that the participants used in daily life was not evaluated in this study. Therefore, the sole type may have influenced the results. Third, the small number of cases limited our study’s potential to detect trends for most results. Although increases in %Long and %Trans were observed, no significant group-by-time interaction effect was found. An adequate number of cases should be included for sufficient statistical power in future research. Furthermore, when the foot pressure tests were performed, the participants used shoes with pressure-sensing insoles provided by the investigator. Participants did not wear their usual shoes or experimental insoles. Therefore, the test results do not completely reflect the patients’ routine usage status. In future studies, patients should wear their usual shoes, and pressure-sensing insoles should be placed under the experimental insoles to obtain data close to routine use. Moreover, foot pain and ankle conditions must be considered. For example, Saito et al. [2] observed different outcomes in patients with knee OA with or without foot pain after total knee arthroplasty. In addition, finite-element-based computational approaches can be considered in future study designs to reduce the cost and obtain a detailed plantar stress distribution through the model [30,31].

## 5. Conclusions

For patients with knee OA, lateral-wedge insoles can prevent the deterioration of plantar-pressure patterns. In this study, the lateral-wedge insole group had stable plantar pressures, while the ordinary insole group showed a pressure-shifting pattern in the central foot. In addition, wireless pressure-sensing insoles are easy to wear, not limited to specific scenarios, and provide an objective assessment of the walking function. Thus, wireless pressure-sensing insoles offer several advantages for such experiments and daily activity monitoring. Further research should consider lower limb biomechanics and different orthosis designs.

## Figures and Tables

**Table 1:** Demographic characteristics of the patients.

	Lateral-Wedge Insole (N = 13)	Ordinary Insole (N = 10)	*p* Value
Age (years)	70.0 ± 4.5 (67.3–72.7)	67.9 ± 8.8 (61.6–74.2)	0.462
Female (number, %)	8 (61.5)	9 (90.0)	0.132
Body weight (kg)	61.5 ± 9.7 (55.6–67.4)	57.9± 9.2 (51.4–64.5)	0.382
Body Mass Index (kg/m^2^)	24.3 ± 2.2 (23.0–25.7)	24.4± 3.1 (22.2–26.6)	0.928

**Table 2:** Visual Analog Scale obtained at weeks 0 and 20.

	Lateral-Wedge Insole	Ordinary Insole	Group by Time (*p*)
Week 0	3.2 ± 2.1 (2.0–4.5)	4.2 ± 1.9 (2.8–5.5)	0.718
Week 20	3.0 ± 2.1 (1.7–4.3)	3.7 ± 1.8 (2.4–5.0)	

**Table 3:** The data of WOMAC at weeks 0 and 20.

	Lateral-Wedge Insole	Ordinary Insole	Group by Time (*p*)
Pain (0–20)			
Week 0	5.0 ± 3.4 (3.0–7.0)	6.8 ± 3.3 (4.4–9.2)	0.622
Week 20	4.2 ± 3.2 (2.2–6.1)	5.3 ± 3.7 (2.6–8.0)	
Stiffness (0–8)			
Week 0	2.1 ± 1.7 (1.1–3.1)	3.3 ± 2.5 (1.5–5.1)	0.423
Week 20	2.1 ± 1.6 (1.1–3.0)	2.8 ± 2.2 (1.2–4.4)	
Function (0–68)			
Week 0	14.2 ± 9.2 (8.7–19.8)	24.8 ± 15.8 (13.5–36.1)	0.031 *
Week 20	14.9 ± 11.3 (8.0–21.7)	16.5 ± 13.2 (7.1–25.9) ^a^	
Total (0–96)			
Week 0	21.3 ± 13.5 (13.1–29.5)	34.9 ± 20 (20.6–49.2)	0.053
Week 20	21.1 ± 15.8 (11.5–30.6)	24.6 ± 18 (11.7–37.5) ^b^	

Mean ± SD (95% confidence interval); * indicated *p* < 0.05. ^a,b^ significant difference between weeks 0 and 20 in the same group. Higher scores indicated higher severity.

**Table 4:** The center of pressure displacement during quiet standing at weeks 0 and 20.

	Lateral-Wedge Insole	Ordinary Insole	Group by Time (*p*)
AP displacement			
Week 0	29.7 ± 21.3 (16.8–42.5)	24.7 ± 12.6 (15.7–33.8)	0.062
Week 20	34.8 ± 29.5 (17.0–52.7)	56.9 ± 31.5 (34.3–79.4) ^a^	
ML displacement			
Week 0	32.1 ± 39.2 (8.4–55.8)	31.6 ± 25.3 (13.5–49.7)	0.720
Week 20	23.7 ± 21.2 (10.9–36.6)	29.8 ± 26.1 (11.1–48.5)	

Unit of displacement: millimeter; AP: anteroposterior; ML: mediolateral; ^a^ significant difference between weeks 0 and 20 in the same group.

**Table 5:** The percentage of the COP path at weeks 0 and 20.

	**Lateral-Wedge Insole**	**Ordinary Insole**	**Group by Time (*p*)**
%Long			
Week 0	13.1 ± 16.0 (3.4–22.8)	21.3 ± 21.2 (6.2–36.5)	0.724
Week 20	24.2 ± 12.9 (16.4–32.0)	36.3 ± 20.8 (21.5–51.2)	
%Trans			
Week 0	3.9 ± 5.2 (0.8–7.1)	6.3 ± 6. 9 (1.4–11.3)	0.400
Week 20	9.2 ± 6.4 (5.3–13.0)	14.8 ± 11.1 (6.9–22.8) ^a^	

COP: center of pressure; %Long: percentage of the anteroposterior length of the COP path to the foot length; %Trans: percentage of the transverse width of the COP path to the maximum foot width. ^a^ significant difference between weeks 0 and 20 in the same group

**Table 6:** Partial foot pressure per body weight at weeks 0 and 20 (unit: %).

	Lateral-Wedge Insole	Ordinary Insole	Group by Time (*p*)
Hallux			
Week 0	8.9 ± 3.1 (7.0–10.7)	8.7 ± 2.4 (7.0–10.4)	0.654
Week 20	7.8 ± 3.3 (5.8–9.8)	6.9 ± 4.7 (3.6–10.3)	
Lateral toes			
Week 0	4.8 ± 2.8 (3.1–6.5)	3.2 ± 2.9 (1.1–5.3)	0.057
Week 20	4.4 ± 4.3 (1.8–7.0)	8.8 ± 8.6 (2.6–14.9)	
Forefoot			
Week 0	31.5 ± 5.5 (28.2–34.8)	30.3 ± 5.8 (26.1–34.4)	0.031 *
Week 20	30.1 ± 11.9 (22.9–37.3)	39.2 ± 8.5 (33.1–45.3) ^a^	
Medial midfoot			
Week 0	8.5 ± 4.2 (5.9–11.0)	7.3 ± 5.5 (3.3–11.2)	0.520
Week 20	9.0 ± 4.7 (6.1–11.8)	10.2 ± 10.0 (3.0–17.4)	
Lateral midfoot			
Week 0	21.2 ± 4.2 (18.6–23.7)	22.5 ± 5.4 (18.6–26.3)	0.154
Week 20	21.4 ± 7.5 (16.9–26.0)	18.0 ± 7.1 (12.9–23.1)	
Heel			
Week 0	25.2± 6.3 (21.3–29.0)	28.1 ± 4.6 (24.8–31.4)	0.024 *
Week 20	27.3 ± 10.5 (21.0–33.7)	16.9 ± 11.4 (8.8–25.0) ^b^	

Mean ± SD (95% confidence interval); * indicated *p* < 0.05. ^a,b^ significant difference between weeks 0 and 20 in the same group.

## Data Availability

The data presented in this study are available on request from the corresponding author.

## Supplementary Materials

The following supporting information can be downloaded at: https://www.mdpi.com/article/10.3390/s23010084/s1, Section S1: Equations for calculating pressure sensing data.; Table S1: The specifications of the pressure sensing insole.
