# Supplementary material for: A Study on the Effects of Lateral-Wedge Insoles on Plantar-Pressure Pattern for Medial Knee Osteoarthritis Using the Wearable Sensing Insole
Source: Sensors (Basel). 2022 Dec 22;23(1):84. doi: 10.3390/s23010084 (PMC9824433; doi:10.3390/s23010084)
Supplement: Supplementary file 1 [file sensors-23-00084-s001.zip › sensors-2046449-supplementary.pdf]

Supplementary Materials

A study on the effects of lateral wedge insoles on plantar pressure pattern for medial knee osteoarthritis using the wearable sensing insole

Wei-Ching Hsu[1,2], Li-Wei Chou[2,3,4], Hsiao-Yen Chiu[1], Chang-Wei Hsieh[5,*], Wen-Pin Hu[1,*]

[1] Department of Bioinformatics and Medical Engineering, Asia University, Taichung City 41354, Taiwan

[2] Department of Physical Medicine and Rehabilitation, Asia University Hospital, Taichung City 41354, Taiwan

[3] Department of Physical Medicine and Rehabilitation, China Medical University Hospital, Taichung City 40402, Taiwan

[4] Department of Physical Therapy, Graduate Institute of Rehabilitation Science, China Medical University, Taichung City 40402, Taiwan

[5] Computer Science & Information Engineering, Asia University, Taichung City 41354, Taiwan

* Correspondence: wenpinhu@asia.edu.tw (W.-P. H.); cwhsien@asia.edu.tw (C.-W. H.)

S1 Equations for calculating pressure sensing data

S1.1 The center of pressure displacement

Matlab (MathWorks, Inc., Natick, MA, USA) was adopted to write programs to analyze the data acquired from insoles. The center of pressure (COP) on the plantar surface and the COP of the body are estimated from the recorded data. The weighted-mean approach was used to calculate the COP trajectories of both feet. The equations have been presented in the paper reported by our group [1]. The pressure-sensing insole coordinate system is defined (shown in Figure 3 of this report [1]). The subjects were asked to stand quietly at the hip-width stance and the distance between the two heels was measured to apply in giving the coordinate values to the sensing spots. The coordinate values of sensing spots should be adjusted by the distance between the two heels. Then, the foot plantar COPs in the medial-lateral (ML) (COP_{px}) and the anterior-posterior (AP) (COP_{py}) directions were calculated according to Eqs. (S1) and (S2).

$$COP_{px} = \frac{\sum C_i^x F_i}{F_{total}} \qquad \text{(S1)}$$

$$COP_{py} = \frac{\sum C_i^y F_i}{F_{total}} \qquad \text{(S2)}$$

where C_i^x and C_i^y denote the corresponding x- and y-coordinate values of sensing spots defined in the coordinate system, respectively. F_i is the value of the pressure sensing spot, and F_{total} is the sum of the values of sensing spots on the insole. Eqs. (S1) and (S2) can apply to calculate the COP of each foot. Therefore, the path of COP for each foot can be obtained, which is used in the calculation of the COP of a body in quiet standing by using Eqs. (S3) and (S4).

The center-of-pressure path of a subject in the medial-lateral (COP_x) and the anterior-posterior (COP_y) directions were computed in light of Eqs. (S3) and (S4).

$$COP_x = \frac{COP_{R,px} \times F_{R,total} + COP_{L,px} \times F_{L,total}}{F_{R,total} + F_{L,total}} \qquad \text{(S3)}$$

$$COP_y = \frac{COP_{R,py} \times F_{R,total} + COP_{R,py} \times F_{L,total}}{F_{R,total} + F_{L,total}} \qquad \text{(S4)}$$

where $COP_{R,px}$ and $COP_{L,px}$ represent the coordinate values of COP in the medial-lateral direction from the insoles of the right and left feet, respectively. $COP_{R,py}$ and $COP_{L,py}$ are the coordinate values of COP in the AP direction obtained from the two insoles on feet. $F_{R,total}$ is the sum of all values measured by sensors on the insole of right foot, and $F_{L,total}$ is used for expressing the sum from the left foot.

S1.2 The percentage of the COP path

%Long: percentage of the anteroposterior length of the COP path to the foot length

$$\%\text{Long} = \frac{The\ anteroposterior\ length\ of\ the\ COP\ path}{Foot\ length} \qquad \text{(S5)}$$

%Trans: percentage of the transverse width of the COP path to the maximum foot width

$$\%\text{Trans} = \frac{The\ transverse\ width\ of\ the\ COP\ path}{Foot\ width} \times 100 \qquad \text{(S6)}$$

S1.3 The percentages of pressure distribution during walking

$$\text{The percentages of pressure distribution} = \frac{F_{sum}}{F_{total}} \times 100 \qquad \text{(S7)}$$

where F_{sum} is the sum of the values of sensors within the defined area, and the F_{total} is the sum of the values of sensors on the insole.

References

1. Chou L.-W.; Shen J.-H.; Lin H.-T.; Yang Y.-T.; Hu W.-P. A Study on the Influence of Number/Distribution of Sensing Points of the Smart Insoles on the Center of Pressure Estimation for the Internet of Things Applications. *Sustainability* **2021**, *13*, 2934.

Table. S1. The specifications of the pressure sensing insole

Specifications of flexible pressure insole

**Number of sensors**	89
**Diameter of each sensor**	1 cm
**Pattern of sensor**	Circle
**Thickness of sensor**	< 300 μm
**Measurable Range**	0 ~ 140 psi<br>Linear Range: 0 ~ 40 psi, corresponding output value between 0 and 255
**Resolution**	2% full scale
**Hysteresis**	±8% full scale
**Response time**	<2 milliseconds
**Scanning frequency**	~30 Hz
**Signal transmission**	Bluetooth
**Size of control box**	50 mm × 80 mm, Thickness < 20 mm
